# Prognostic effects of glycaemic variability on diastolic heart failure and type 2 diabetes mellitus: insights and 1-year mortality machine learning prediction model

**DOI:** 10.1186/s13098-024-01534-2

**Published:** 2024-11-23

**Authors:** Zhenkun Yang, Yuanjie Li, Yang Liu, Ziyi Zhong, Coleen Ditchfield, Taipu Guo, Mingjuan Yang, Yang Chen

**Affiliations:** 1 Department of Cardiology, Tianjin Medical University General Hospital, Tianjin, China; 2 Tianjin Research Institute of Anesthesiology, Department of Anesthesiology, Tianjin Medical University General Hospital, Tianjin, China; 3 Department of Cardiovascular Medicine, the Second Affiliated Hospital, Jiangxi Medical College, Nanchang University, Nanchang, Jiangxi People’s Republic of China; 4 Liverpool Centre for Cardiovascular Science, University of Liverpool, Liverpool John Moores University and Liverpool Heart and Chest Hospital, Liverpool, UK; 5 Department of Musculoskeletal Ageing and Science, Institute of Life Course and Medical Sciences, University of Liverpool, Liverpool, UK; 6 Department of Medicine for Older People, Whiston Hospital, Mersey and West Lancashire Teaching Hospitals NHS Trust, Prescot, UK; 7 Department of Cardiovascular and Metabolic Medicine, Institute of Life Course and Medical Sciences, University of Liverpool, Liverpool, UK

**Keywords:** Glycaemic variability, Heart failure with preserved ejection fraction, Type 2 diabetes mellitus, Mortality, Machine learning

## Abstract

**Background:**

Diastolic heart failure (DHF) and type 2 diabetes mellitus (T2DM) often coexist, causing increased mortality rates. Glycaemic variability (GV) exacerbates cardiovascular complications, but its impact on outcomes in patients with DHF and T2DM remains unclear. This study examined the relationships between GV with mortality outcomes, and developed a machine learning (ML) model for long-term mortality in these patients.

**Methods:**

Patients with DHF and T2DM were included from the Medical Information Mart for Intensive Care IV, with admissions (2008–2019) as primary analysis cohort and admissions (2020–2022) as external validation cohort. Multivariate Cox proportional hazards models and restricted cubic spline analyses were used to evaluate the associations of GV with 90-day, 1-year, and 3-year all-cause mortality. The primary analysis cohort was split into training and internal validation cohorts, then developing ML models for predicting 1-year all-cause mortality in training cohort, which were validated using the internal and external validation cohorts.

**Results:**

2,128 patients with DHF and T2DM were included in primary analysis cohort (meidian age 71.0years [IQR: 62.0–79.0]; 46.9% male), 498 patients with DHF and T2DM were included in the external validation cohort (meidian age 75.0years [IQR: 67.0–81.0]; 54.0% male). Multivariate Cox proportional hazards models showed that high GV tertiles were associated with higher risk of 90-day (T2: HR 1.45, 95%CI 1.09–1.93; T3: HR 1.96, 95%CI 1.48–2.60), 1-year (T2: HR 1.25, 95%CI 1.02–1.53; T3: HR 1.54, 95%CI 1.26–1.89), and 3-year (T2: HR 1.31, 95%CI: 1.10–1.56; T3: HR 1.48, 95%CI 1.23–1.77) all-cause mortality, compared with lowest GV tertile. Chronic kidney disease, creatinine, potassium, haemoglobin, and white blood cell were identified as mediators of GV and 1-year all-cause mortality. Additionally, GV and other clinical features were pre-selected to construct ML models. The random forest model performed best, with AUC (0.770) and G-mean (0.591) in internal validation, with AUC (0.753) and G-mean (0.599) in external validation.

**Conclusion:**

GV was determined as an independent risk factor for short-term and long-term all-cause mortality in patients with DHF and T2DM, with a potential intervention threshold around 25.0%. The ML model incorporating GV demonstrated strong predictive performance for 1-year all-cause mortality, highlighting its importance in early risk stratification management of these patients.

**Supplementary Information:**

The online version contains supplementary material available at 10.1186/s13098-024-01534-2.

## Introduction

Heart failure with preserved ejection fraction (DHF) represents a significant proportion of heart failure (HF) cases, with DHF incidence increasing markedly with age [1]. Large-scale clinical trials have demonstrated that DHF patients experience an average of 1.4 hospitalizations per year and face an annual mortality rate of approximately 15%.^2,3^ The global prevalence of type 2 diabetes mellitus (T2DM) continues to rise, with around 537 million adults (aged 20–79 years) diagnosed in 2021, a number projected to reach 783 million by 2045 [4]. Mortality rates are significantly higher in T2DM patients compared to non-diabetic individuals, particularly when accompanied by cardiovascular disease. T2DM also increases the risk of all-cause mortality and 30-day readmission for HF [5]. The coexistence of DHF and T2DM is common, with 30–40% of HF patients also having diabetes, a figure that can rise to 50% among hospitalized patients [6]. 

Glycaemic variability (GV) refers to the fluctuations in blood glucose levels over time, capturing the dynamic changes in an individual’s blood glucose levels. Factors such as incorrect insulin dosage and timing, irregular diet and exercise, psychological stress, emotional fluctuations, and certain medications can all influence GV [7]. High GV is associated with increased oxidative stress, which is more damaging to blood vessels and tissues than sustained hyperglycemia [8, 9]. This oxidative stress triggers inflammatory responses, leading to a chronic inflammatory state that can impair endothelial function, contributing to atherosclerosis and cardiovascular diseases (CVD) [10, 11]. GV also increases vascular stiffness, which has been linked to higher incidence and mortality rates in cardiovascular conditions like coronary artery disease and HF [12–14]. Additionally, GV is closely associated with the progression of diabetic complications, including retinopathy and neuropathy [15, 16]. Moreover, high GV exacerbates glomerular pressure and oxidative stress, leading to glomerular basement membrane thickening and glomerulosclerosis, which in turn impair renal function [16–18]. Furthermore, GV is linked to more severe cerebrovascular damage and cognitive decline [19], as well as non-alcoholic fatty liver disease [20], and an increased risk of adverse outcomes in cancer [21]. In HF patients, GV exacerbates myocardial fibrosis and ventricular remodeling, further worsening their condition [22]. These patients are more prone to acute cardiac events in the short term and face a poorer long-term prognosis [16]. Frequent episodes of hypoglycemia and hyperglycemia also severely impact their quality of life and mental health. However, the relationship between GV and prognosis in patients with heart failure with DHF and T2DM remains unexplored.

Given the high risk of long-term mortality in patients with DHF and T2DM, there is currently no established prediction model tailored to this population. Traditional risk scoring systems often fall short in capturing the complex, multidimensional risk factors associated with these coexisting conditions. Machine learning offers a promising approach for developing more accurate prediction models, particularly in the field of cardiovascular disease [23]. Therefore, creating a machine learning-based model to predict long-term mortality in patients with DHF and T2DM is essential for improving clinical outcomes and guiding treatment strategies.

Therefore, The aims of this study are twofold. (1) To investigate the relationship between GV and outcomes in DHF and T2DM patients. (2) To evaluate whether GV can be used to construct a ML prediction model for long-term all-cause mortality in DHF and T2DM. These could help clinicians identify high-risk individuals and improve preventive screening and treatment strategies.

## Methods

### Data sources

The original data for this study were sourced from the Medical Information Mart for Intensive Care IV (MIMIC-IV, version 3.0), which contains detailed records of ICU hospitalizations at Beth Israel Deaconess Medical Centre (BIDMC) from 2008 to 2022 [24]. Ethical approval for data collection was granted by the institutional review boards of the BIDMC (2001-P-001699/14) and Massachusetts Institute of Technology (No.0403000206). In this analysis, all data were anonymised and could therefore be exempt from local ethics review committee. The author, ZKY, had access to the database and extracted all the data for this study (No.57121385).

### Study participants

We included all patients diagnosed with DHF and T2DM based on International Classification of Diseases version 10 (ICD-10) [DHF: I50.3; T2DM: E11]. Exclusion criteria were as following: (1) fewer than three blood glucose measurements during hospitalisation; (2) age under 17 years; (3) survival time or length of hospital stay less than 24 h; and (4) records of multiple hospitalizations.

### Extracted covariates

We extracted the following covariates: demographics variables (age, sex and race), comorbidities (coronary artery disease [CAD], chronic obstructive pulmonary disease [COPD], myocardial infarction [MI], atrial fibrillation [AF], chronic kidney disease [CKD], stroke, cardiomyopathy, dyslipidemia, hypertension, hepatic disease, malignant cancer), laboratory results (creatinine, potassium, calcium, hemoglobin, platelet, white blood cell count [WBC], red blood cell distribution width [RDW]), medications (diuretic, insulin, angiotensin converting enzyme inhibitors [ACEI]/angiotensin receptor blocker [ARB], beta-blocker, statin, antiplatelet agent, anticoagulant), and interventions (coronary artery bypass grafting, percutaneous coronary intervention, implantable cardioverter defibrillator pacemaker). Of these, diuretic included furosemide and spironolactone; antiplatelet agent included aspirin, clopidogrel, ticlopidine, dipyridamole; anticoagulant included heparin, coumadin, bivalirudin, argatroban, fondaparinux; statin included rosuvastatin, atorvastatin, pitavastatin, fluvastatin, simvastatin. Laboratory tests were analyzed using only the first value recorded on the first-day after admission.

### GV assessment

Blood glucose values were collected at least three times during the hospital stay. GV was quantified using the coefficient of variation, calculated as the ratio of the standard deviation to the mean of these measurements [25]. 

### Study outcomes

Our primary outcome was all-cause mortality at 90-day, 1-year, and 3-year post-admission. Our secondary outcome was the prolonged hospital stay, with a duration of ≥ 11.42 days (75th percentile of the overall cohort) considered prolonged.

### Statistical analysis

Regarding missing data, as shown in Supplementary Table S1, the maximum percentage of missing values was approximately 25%. We used the ‘miceforest’ package in R for multiple imputation to handle these missing values.

For continuous variables, the mean ± standard deviation or the median and interquartile range (IQR) were reported, depending on the data distribution. Group differences were analyzed using one-way analysis of variance or Kruskal-Wallis tests, as appropriate. Differences in categorical variables among groups were assessed using Fisher’s exact test or Chi-squared test, and results were expressed as counts and percentages.

We divided GV into three tertiles: T1 (GV ≤ 19.5%), T2 (19.5% < GV ≤ 30.8%), and T3 (GV > 30.8%). We used Cox proportional hazards model to evaluate the associations between GV with 90-day, 1-year, and 3-year all-cause mortality after hospital admission in patients with DHF and T2DM, reporting hazard ratios (HR) with 95% confidence intervals (95% CI). And restricted cubic spline (RCS) analysis was also used to assess the relationship between GV and mortality outcomes. Moreover, logistic regression model was applied to assess the impact of GV on length of stay, with results presented as odds ratios (OR) with 95% CI. To avoid confounders that could potentially influence the outcome, we adjusted for several different combinations. Model 1: unadjusted; Model 2: adjusted for age, sex, race; Model 3: adjusted for Model 2 and comorbidities (CAD, COPD, MI, AF, CKD, stroke, cardiomyopathy, dyslipidemia, hypertension, hepatic disease, malignant cancer). Model 4: adjusted for Model 3 and laboratory results (creatinine, potassium, calcium, hemoglobin, platelet, WBC, RDW), medications (diuretic, insulin, ACEI/ARB, beta-blocker, statin, antiplatelet agent, anticoagulant), and interventions (coronary artery bypass grafting, percutaneous coronary intervention, implantable cardioverter defibrillator pacemaker). We conducted variance inflation factor testing on the covariates included in Model 4 to assess multicollinearity and ensure model validity, all variance inflation factors of covariates were less than 10 (Supplementary Table S2).

Kaplan-Meier survival curves were used to compare survival distributions across the GV tertiles, with log-rank tests evaluating differences between the groups. Subgroup analyses were then performed based on age (≥ 65 or < 65 years), sex, and CKD to assess the consistency of the associations between GV with 90-day, 1-year, and 3-year all-cause mortality post-admission in patients with DHF and T2DM.

Based on the survival trends observed in the Kaplan-Meier curves across GV tertile groups, we used the ‘survminer’ package in R to determine the optimal cut-off point of GV. This threshold allows for more precise risk stratification of mortality in patient with DHF and T2DM, thereby providing clearer guidance for clinical decision-making.

Moreover, we conducted several sensitivity analyses. First, patients who died during their hospital stay were likely critically ill at admission, suggesting that their condition, rather than GV, might have contributed to their mortality, raising the possibility of reverse causality. To mitigate this, we excluded in-hospital deaths and further analyzed the relationships between GV with 90-day, 1-year, and 3-year all-cause mortality post-discharge in patients with DHF and T2DM. In this post-discharge analysis, GV was categorized into three tertiles: T1-post-discharge (GV ≤ 18.9%), T2-post-discharge (18.9% < GV ≤ 30.6%), and T3-post-discharge (GV > 30.6%). Second, the association between GV and the risk of mortality outcomes may be affected by blood glucose at admission, mean blood glucose during hospitalization, and long-term blood glucose control (as represented by hemoglobinA1c [HbA1c]). Therefore, we further added admission blood glucose level, mean blood glucose level during hospitalization, and HbA1c to Model 4 to test the stability of the results. Third, we excluded patients with malignant cancer to assess the robustness of the association, as these patients have a shorter expected lifespan that could influence the primary outcomes. Fourth, we aimed to evaluate the robustness of GV as an indicator by using alternative measures: standard deviation (SD) and variability independent of the mean (VIM). SD reflects the overall fluctuation around the mean blood glucose during the hospital stay, while VIM standardizes variability by adjusting for the mean blood glucose, calculated as VIM = SD / mean^1.5^. This approach ensures that the impact of GV on clinical outcomes is assessed consistently, accounting for baseline glucose differences across patients and providing a more stable evaluation of blood glucose fluctuations.

To further assess the potential of GV to enhance existing predictive models, we selected the 3A3B score as a baseline model for predicting outcomes in DHF patients [26]. The 3A3B score was chosen for its simplicity and ease of application, as it includes variables that are readily available and more feasible to capture in MIMIC IV. We then evaluated whether adding GV improved the model’s performance by calculating the Net Reclassification Improvement (NRI) and Integrated Discrimination Improvement Index (IDI).

Given the absence of a long-term mortality prediction model for patients with DHF and T2DM, and the fact that the *external validation cohort* only included 1-year all-cause mortality outcome, we aimed to develop a ML model to predict 1-year all-cause mortality in this population. First, the 2008–2019 cohort was split into *training and internal validation cohorts* in a 7:3 ratio, and Boruta was used for feature pre-selection in the *training cohort*. Next, Pearson correlation and variance inflation factor tests were performed to ensure no strong correlations or multicollinearity between the selected features. Then, the selected features were introduced into seven commonly used ML algorithms, including light gradient boosting machine (LightGBM), random forest (RF), logistic regression, support vector machine, multilayer perceptron, Gaussian Naive Bayes, and K-nearest neighbors. The performance of these models was evaluated using receiver operating characteristic (ROC) curves and decision curve analysis (DCA) in both the *internal and external validation cohorts*. Metrics such as the area under the curve (AUC), accuracy, specificity, precision, recall, F1-score, and G-mean were calculated. The best-performing ML model was selected based on these criteria. Finally, SHapley Additive exPlanations (SHAP) values were calculated to assess these features importance, with dependent SHAP plots specifically generated for GV to illustrate its correlation with 1-year all-cause mortality in both the *internal and external validation cohorts*.

To assess the mediators between the GV and 1-year all-cause mortality, we performed a mediation analysis using the “mediation” package in R. This package allows for the estimation of causal mediation effects, enabling to explore the indirect pathways through which GV influences 1-year all-cause mortality in patients with DHF and T2DM.

All analyses were performed using SPSS Statistics (version 27, USA), R (version 4.3.2, Austria), and Python (version 3.11.1, USA). Two-tailed *P* < 0.05 was considered statistically significant.

## Results

### Baseline characteristics

Final, 2,182 patients with DHF and T2DM were included in the primary analysis cohort (Supplementary Fig. S1). Table 1 presents the baseline characteristics of primary analysis cohort, stratified by tertiles of GV. The median age of the primary cohort was 71.0 years (IQR: 62.0–79.0), and 1024 patients (46.9%) were male. Patients in the T3 had significantly more comorbidities, including CAD (*P* = 0.008), MI (*P* = 0.003), AF (*P* < 0.001), CKD (*P* < 0.001), and stroke (*P* = 0.029). Additionally, they had higher levels of creatinine (*P* < 0.001), potassium (*P* < 0.001), and WBC (*P* < 0.001), with a greater proportion receiving insulin (*P* < 0.001) and antiplatelet agents (*P* = 0.014).

**Table 1:** Baseline characteristics according to tertiles of glycemic variability

Characteristics	Overall (*N* = 2182)	T1 (*N* = 725)	T2 (*n* = 727)	T3 (*n* = 730)	*P*
Age, years	71 (62–79)	72 (64–81)	72 (63–80)	70 (61–77)	< 0.001
Male, n (%)	1024 (46.9%)	330 (45.5%)	330 (45.4%)	364 (49.9%)	0.150
Race, n (%)					0.099
Black	404 (18.5%)	111 (15.3%)	147 (20.2%)	146 (20.0%)	
White	1370 (62.8%)	472 (65.1%)	452 (62.2%)	446 (61.1%)	
Others	408 (18.7%)	142 (19.6%)	128 (17.6%)	138 (18.9%)	
Comorbidities, n (%)					
Coronary artery disease	978 (44.8%)	292 (40.3%)	335 (46.1%)	351 (48.1%)	0.008
COPD	540 (24.7%)	168 (23.2%)	182 (25.0%)	190 (26.0%)	0.440
Myocardial infarction	304 (13.9%)	79 (10.9%)	100 (13.8%)	125 (17.1%)	0.003
Atrial fibrillation	980 (44.9%)	365 (50.3%)	325 (44.7%)	290 (39.7%)	< 0.001
Chronic kidney disease	1093 (50.1%)	316 (43.6%)	377 (51.9%)	400 (54.8%)	< 0.001
Stroke	311 (14.3%)	108 (14.9%)	84 (11.6%)	119 (16.3%)	0.029
Cardiomyopathy	129 (5.9%)	46 (6.3%)	37 (5.1%)	46 (6.3%)	0.515
Dyslipidemia	1318 (60.4%)	448 (61.8%)	428 (58.9%)	442 (60.5%)	0.521
Hypertension	189 (8.7%)	68 (9.4%)	63 (8.7%)	58 (7.9%)	0.623
Hepatic disease	185 (8.5%)	50 (6.9%)	68 (9.48%)	67 (9.2%)	0.172
Malignant cancer	252 (11.5%)	87 (12.0%)	80 (11.0%)	85 (11.6%)	0.834
Laboratory results					
Creatinine, mg/dL	1.40 (1.00-2.30)	1.20 (0.90–1.90)	1.40 (1.00-2.50)	1.50 (1.10–2.50)	< 0.001
Potassium, mmol/L	4.30 (3.90–4.70)	4.20 (3.90–4.70)	4.30 (3.90–4.70)	4.40 (4.00-4.80)	< 0.001
Calcium, mg/dL	8.80 (8.30–9.20)	8.80 (8.40–9.20)	8.80 (8.30–9.20)	8.80 (8.30–9.20)	0.271
Hemoglobin, g/dL	10.00 (8.60–11.60)	10.30 (8.80–11.90)	9.80 (8.50–11.50)	9.90 (8.60–11.40)	0.001
Platelet, ×10^^9^/L	203.00 (153.00-257.00)	199.00 (151.50–252.00)	203.00 (153.00-255.00)	207.00 (155.00-266.25)	0.187
WBC, ×10^^9^/L	8.70 (6.60–11.80)	8.20 (6.40–11.30)	8.70 (6.70–11.70)	9.20 (6.80-12.43)	< 0.001
RDW, %	15.10 (13.90-16.82)	15.00 (13.90–16.50)	15.30 (14.00-17.10)	15.20 (13.90-16.82)	0.034
Treatments, n (%)					
Diuretic	1805 (82.7%)	580 (80.0%)	607 (83.5%)	618 (84.7%)	0.050
Insulin	1910 (87.5%)	559 (77.1%)	650 (89.4%)	701 (96.0%)	< 0.001
ACEI/ARB	662 (30.3%)	221 (30.5%)	204 (28.1%)	237 (32.5%)	0.187
Beta blocker	1236 (56.6%)	389 (53.7%)	418 (57.5%)	429 (58.8%)	0.123
Statin	1542 (70.7%)	502 (69.2%)	511 (70.3%)	529 (72.5%)	0.387
Antiplatelet agent	1387 (63.6%)	445 (61.4%)	447 (61.5%)	495 (67.8%)	0.014
Anticoagulant	896 (41.1%)	320 (44.1%)	306 (42.1%)	270 (37.0%)	0.017
CABG/PCI	58 (2.7%)	16 (2.2%)	16 (2.2%)	26 (3.6%)	0.177
ICD pacemaker	177 (8.1%)	75 (10.3%)	55 (7.6%)	47 (6.4%)	0.019

T1: GV ≤ 19.5%, T2: 19.5% < GV ≤ 30.8%, and T3: GV > 30.8%

Abbreviations: ACEI, angiotensin converting enzyme inhibitor; ARBs, angiotensin receptor blocker; CABG, coronary artery bypass grafting; COPD, chronic obstructive pulmonary disease; ICD, implantable cardioverter defibrillator; PCI, percutaneous coronary intervention; RDW, red blood cell distribution width; WBC, white blood cell count

### Associations of GV and 90-day, 1-year, and 3-year all-cause mortality

**Fig. 1:**
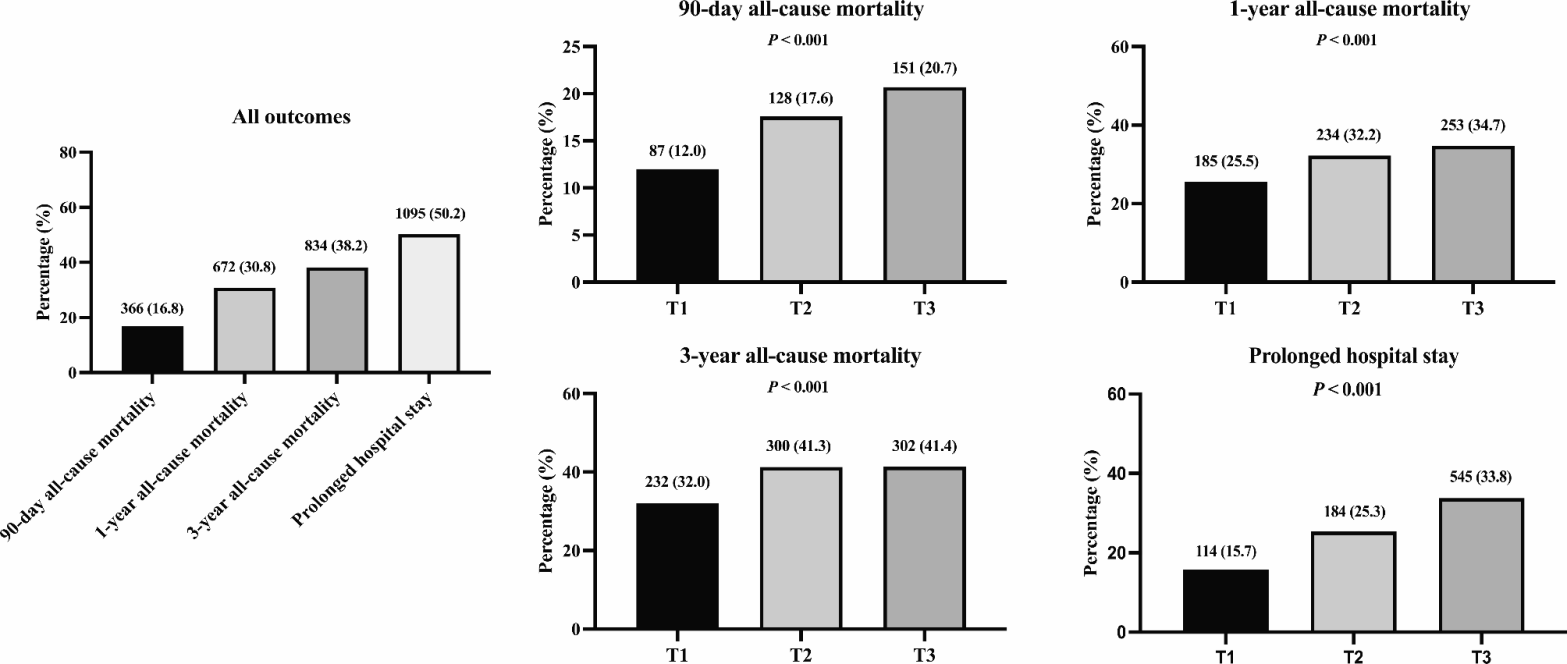
Study outcomes across tertiles of glycaemic variability

**Fig. 2:**
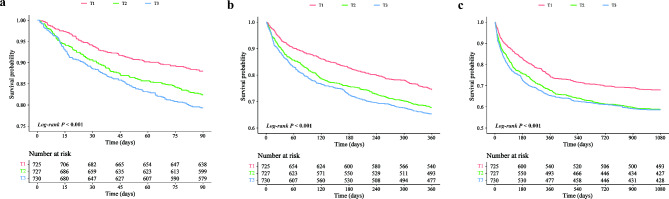
Kaplan-Meier curves for the tertiles of glycaemic variability. **a**: 90-day all-cause mortality; **b**: 1-year all-cause mortality; **c**: 3-year all-cause mortality

**Fig. 3:**
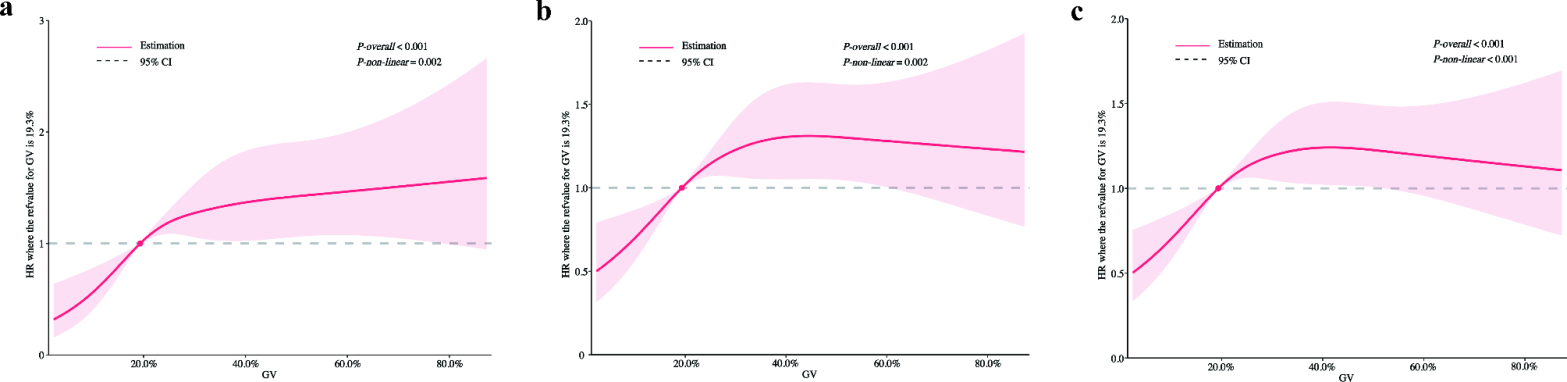
Restricted cubic spline analyses for GV and mortality outcomes. **a**: 90-day all-cause mortality; **b**: 1-year all-cause mortality; **c**: 3-year all-cause mortality. CI, confidence interval; GV, glycaemic variability; HR, hazard ratio

Figure 1 and Supplementary Table S3 illustrate the distribution of outcomes across the GV tertiles. 90-day, 1-year, and 3-year all-cause mortality at progressively increased with order of T1, T2, T3 (90-day: T1 [12.0%] < T2 [17.6%] < T3 [20.7%]; 1-year: T1 [25.5%] < T2 [32.2%] < T3 [34.7%]; 3-year: T1 [32.0%] < T2 [41.3%] < T3 [41.4%]; all *P* < 0.001). Kaplan-Meier curves showed that the survival probability of patients with DHF and T2DM across different tertiles at 90 days, 360 days and 1080 days was significantly different (*log-rank P* < 0.001), and the cumulative mortality rate of T2 and T3 was significantly higher than that of T1 (Fig. 2). The results of the RCS analyses showed that GV was significantly associated with each of the mortality outcomes as well as a significant nonlinear relationship (90-day: *P-overall* < 0.001, *P-non-linear* = 0.002; 1-year: *P-overall* < 0.001, *P-non-linear* = 0.002; 3-year: *P-overall* < 0.001, *P-non-linear* < 0.001) [Fig. 3].

From Table 2, after adjusting for confounders, multivariate Cox proportional hazards models indicated that compared to T1 of GV, T2 and T3 of GV were associated with higher risk of 90-day (T2: HR 1.45, 95% CI 1.09–1.93; T3: HR 1.96, 95% CI 1.48–2.60), 1-year (T2: HR 1.25, 95% CI 1.02–1.53; T3: HR 1.54, 95% CI 1.26–1.89), and 3-year (T2: HR 1.31, 95% CI: 1.10–1.56; T3: HR 1.48, 95% CI 1.23–1.77) all-cause mortality in patients with DHF and T2DM. Additionally, no significant interaction was found for the subgroup analysis of each outcome (Supplementary Fig. S2).

**Table 2:** Associations between GV with 90-day, 1-year, and 3-year all-cause mortality

		Model I		Model II		Model III		Model IV	
		HR (95% CI)	*P*	HR (95% CI)	*P*	HR (95% CI)	*P*	HR (95% CI)	*P*
90-day all-cause mortality*									
	T1	*Reference*		*Reference*		*Reference*		*Reference*	
	T2	1.52 (1.16-2.00)	0.003	1.57 (1.19–2.06)	0.001	1.57 (1.19–2.06)	0.001	1.45 (1.09–1.93)	0.010
	T3	1.82 (1.40–2.37)	< 0.001	2.01 (1.54–2.63)	< 0.001	2.02 (1.55–2.65)	< 0.001	1.96 (1.48–2.60)	< 0.001
1-year all-cause mortality*									
	T1	*Reference*		*Reference*		*Reference*		*Reference*	
	T2	1.34 (1.11–1.63)	0.003	1.39 (1.14–1.68)	< 0.001	1.37 (1.13–1.66)	0.002	1.25 (1.02–1.53)	0.030
	T3	1.48 (1.23–1.79)	< 0.001	1.64 (1.36–1.99)	< 0.001	1.63 (1.34–1.97)	< 0.001	1.54 (1.26–1.89)	< 0.001
3- year all-cause mortality*									
	T1	*Reference*		*Reference*		*Reference*		*Reference*	
	T2	1.39 (1.17–1.65)	< 0.001	1.43 (1.21–1.70)	0.004	1.40 (1.17–1.66)	< 0.001	1.31 (1.10–1.56)	0.003
	T3	1.43 (1.20–1.69)	< 0.001	1.57 (1.32–1.87)	< 0.001	1.54 (1.29–1.83)	< 0.001	1.48 (1.23–1.77)	< 0.001

T1: GV ≤ 19.5%, T2: 19.5% < GV ≤ 30.8%, and T3: GV > 30.8%

Model 1: unadjusted;

Model 2: adjusted for age, sex and race;

Model 3: based on Model 2 further adjusted for coronary artery disease, chronic obstructive pulmonary disease, myocardial infarction, atrial fibrillation, chronic kidney disease; stroke, cardiomyopathy, dyslipidemia, hypertension, hepatic disease, malignant cancer;

Model 4: based on Model 3 further adjusted for creatinine, potassium, calcium, hemoglobin, platelet, white blood cell, red blood cell distribution width, diuretic, insulin, angiotensin converting enzyme inhibitor/angiotensin receptor blocker, beta blocker, statin, antiplatelet agent, anticoagulant, coronary artery bypass grafting/percutaneous coronary intervention, implantable cardioverter defibrillator pacemaker

*P for trend < 0.001 for each model corresponding to this outcome

Abbreviations: CI, confidence interval; GV, glycaemic variability; HR, hazard ratio

### Association of GV and prolonged length of hospital stay

From Fig. 1 & Supplementary Table S3, the proportion of prolonged hospital stay increases with order of T1, T2, T3 (T1 [15.7%] < T2 [25.3%] < T3 [33.8%], *P* < 0.001). Additionally, the results of multivariate logistic regression models indicated that T2 and T3 of GV were related to prolonged length of hospital stay compared with T1 of GV (T2: OR 1.67, 95% CI 1.27–2.21; T3: OR 2.54, 95% CI 1.93–3.34) [Supplementary Table S4].

### Performance comparison of adding GV to previous models

By adding GV to the previous model predicting long-term mortality in DHF, the updated model showed an increase in NRI and IDI of 20.4% and 0.8% for 1-year mortality, and 15.8% and 1.0% for 3-year mortality, respectively, in patients with DHF and T2DM. These results indicate that incorporating GV enhances the model’s ability to discriminate long-term mortality outcomes (Supplementary Table S5).

### Sensitivity analysis

To avoid reverse causality, patients who died within the hospital were excluded. From Supplementary Table S6, multivariate Cox proportional hazards models showed that compared to T1 of GV, T3 of GV was associated with higher risk of 90-day and 1-year all-cause mortality (90-day: HR 1.79, 95% CI 1.29–2.50; 1-year: HR 1.35, 95% CI 1.08–1.69), while T2 showed borderline hazard effects (90-day: HR 1.29; 1-year: HR 1.16). Additionally, T2 and T3 of GV were linked to higher risk of 3-year all-cause mortality compared with T1 of GV (T2: HR 1.25, 95% CI 1.03–1.51; T3: HR 1.34, 95% CI 1.10–1.64).

After including blood glucose at admission, mean blood glucose, and HbA1c in Model 4 to assess the robustness of the effect of GV on the risk of all-cause mortality outcomes. The results were shown that higher GV remained significantly associated with the risk of all-cause mortality outcomes after controlling for these important covariates (for 90-day: T1 as reference, T3: HR 3.00, 95% CI 1.13–7.95; for 1-year: T1 as reference, T3: HR 1.92, 95% CI 1.01–3.63; for 3-year: T1 as reference, T2: HR 1.75, 95% CI: 1.02–3.02; T3: HR 1.79, 95% CI 1.02–3.15) [Supplementary Table S7].

Similarly, after excluding patients with malignant cancer, higher GV was still related to higher risk of all-cause mortality outcomes (for 90-day: T1 as reference, T2: HR 1.63, 95% CI: 1.17–2.27; T3: HR 2.02, 95% CI 1.45–2.83; for 1-year: T1 as reference, T2: HR 1.44, 95% CI: 1.15–1.81; T3: HR 1.69, 95% CI 1.34–2.14; for 3-year: T1 as reference, T2: HR 1.50, 95% CI: 1.22–1.83; T3: HR 1.63, 95% CI 1.33–2.01) [Supplementary Table S8].

The impact on mortality in patients with DHF and T2DM was assessed using SD and VIM, respectively, after adjusting for confounders, multivariate Cox proportional hazards models indicated that compared to T1 of SD, T2 and T3 of SD were associated with higher risk of 90-day (T2: HR 1.70, 95% CI 1.28–2.26; T3: HR 2.04, 95% CI 1.52–2.73), 1-year (T2: HR 1.43, 95% CI 1.17–1.75; T3: HR 1.60, 95% CI 1.30–1.97), and 3-year (T2: HR 1.40, 95% CI: 1.17–1.68; T3: HR 1.52, 95% CI 1.26–1.83); compared to T1 of VIM, T2 and T3 of VIM were associated with higher risk of 90-day (T2: HR 1.49, 95% CI 1.13–1.96; T3: HR 1.61, 95% CI 1.22–2.13), 1-year (T2: HR 1.28, 95% CI 1.05–1.56; T3: HR 1.45, 95% CI 1.19–1.77), and 3-year (T2: HR 1.31, 95% CI: 1.10–1.56; T3: HR 1.38, 95% CI 1.16–1.65) [Supplementary Table S9].

### Optimal cut-off point of GV

**Fig. 4:**
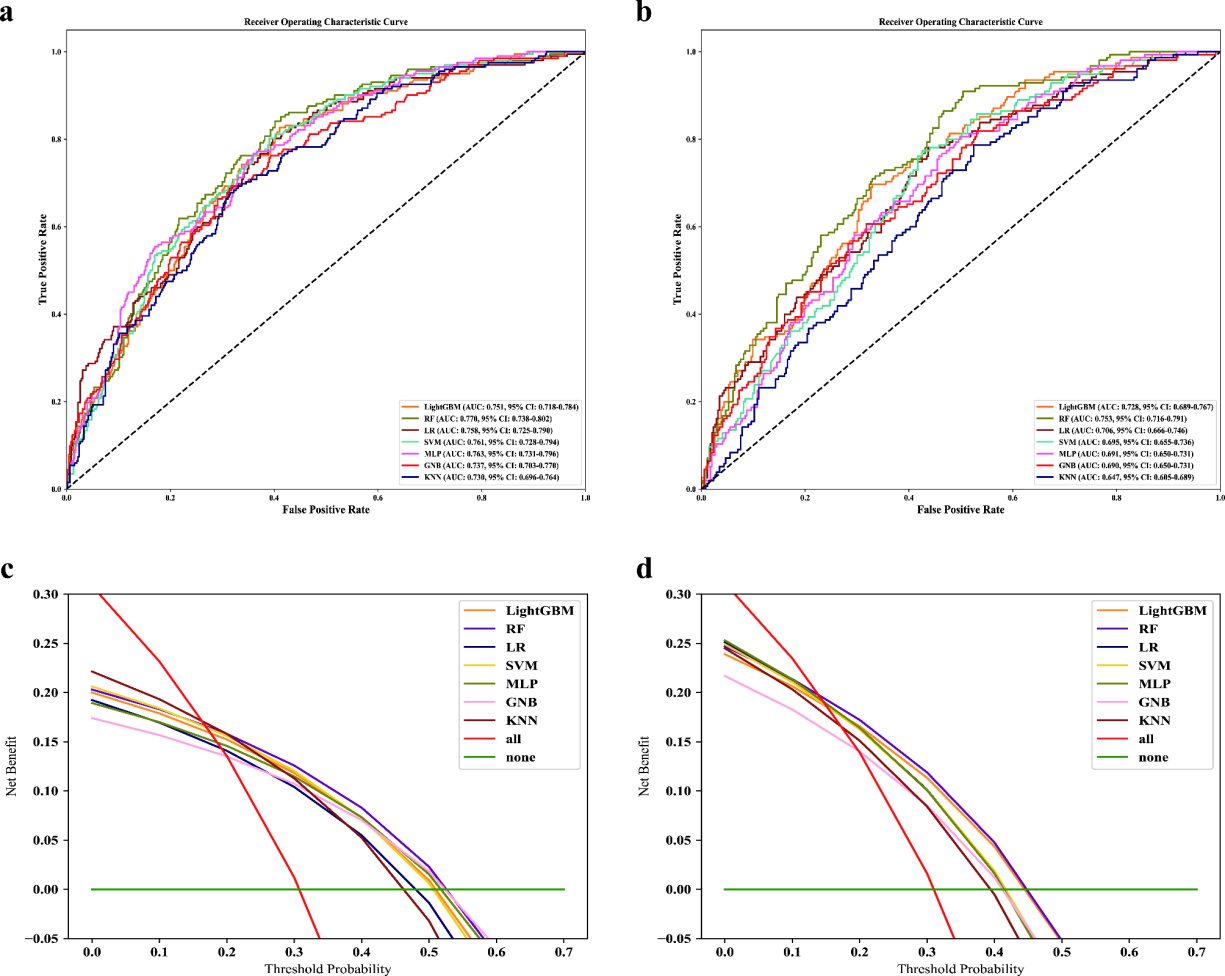
ROC and DCA for machine learning models in the validation cohorts, **a**: ROC in the internal validation; **b**: ROC in the external validation; **c**: DCA in the internal validation; **d**: DCA in the external validation. Red “all” indicates that all patients received treatment, green “none” indicates that all patients did not receive treatment, and the area under each model is the net clinical benefit from applying the predictive model. AUC, area under curve; CI, confidence interval; DCA, decision curve analysis; GNB, Gaussian Naive Bayes; KNN, k-nearest neighbors; LightGBM, light gradient boosting machine; LR, logistic regression; MLP, multilayer perceptron; RF, random forest; ROC, ceceiver operating characteristic curve; SVM, Support Vector Machine

To better stratify the risk of mortality in patients with DHF and T2DM, and to identify targets for clinical intervention, the optimal cut-off points for mortality outcomes were additionally explored. The GV values with the greatest change in curvature of the RCS curves in Fig. 4 were between 20.0% and 30.0%. Subsequently, the cut-off points of GV for 90-day, 1-year, and 3-year all-cause mortality were determined to be 24.1%, 23.2%, and 23.1%, respectively, using the ‘survminer’ package (Supplementary Fig. S3). Considering the simplification of clinical application, the enhancement of operability, and the natural fluctuation of data, the optimal cut-off point for the risk stratification of patients with DHF and T2DM was defined as 25.0%.

From Supplementary Table S10, in the whole primary analysis cohort, the high GV group (GV ≥ 25.0%) had a higher risk of each outcome than the low GV group (GV < 25.0%) [90-day all-cause mortality: HR 1.54, 95% CI 1.24–1.91; 1-year all-cause mortality: HR 1.37, 95% CI 1.17–1.61; 3-year all-cause mortality: HR 1.34, 95% CI 1.16–1.55; prolonged length of hospital stay: OR 1.80, 95% CI 1.45–2.23]. After excluding patients died within the hospital, the high GV group had a higher risk of each outcome than the low GV group (90-day all-cause mortality: HR 1.47, 95% CI 1.13–1.90; 1-year all-cause mortality: HR 1.27, 95% CI 1.06–1.51; 3-year all-cause mortality: HR 1.27, 95% CI 1.08–1.48).

### Model construction

The primary analysis cohort was divided into training and internal validation cohorts by a ratio of 7:3, Boruta was performed in the training cohort (Supplementary Fig. S4). To avoid missing important variables, we included clearly important and uncertain features for 1-year all-cause mortality, including malignant cancer, age, RDW, GV, ACEI/ARB, WBC, platelet, creatinine, AF, potassium, anticoagulant, hemoglobin, antiplatelet agent, calcium, COPD, CKD. There was no strong correlation across these features (Supplementary Fig. S5). These features were then input into seven commonly used ML algorithms in the training cohort. The best hyperparameters for MLs using five-fold cross-validation are shown in Supplementary Table S11.

### Model evaluation and feature importance

**Fig. 5:**
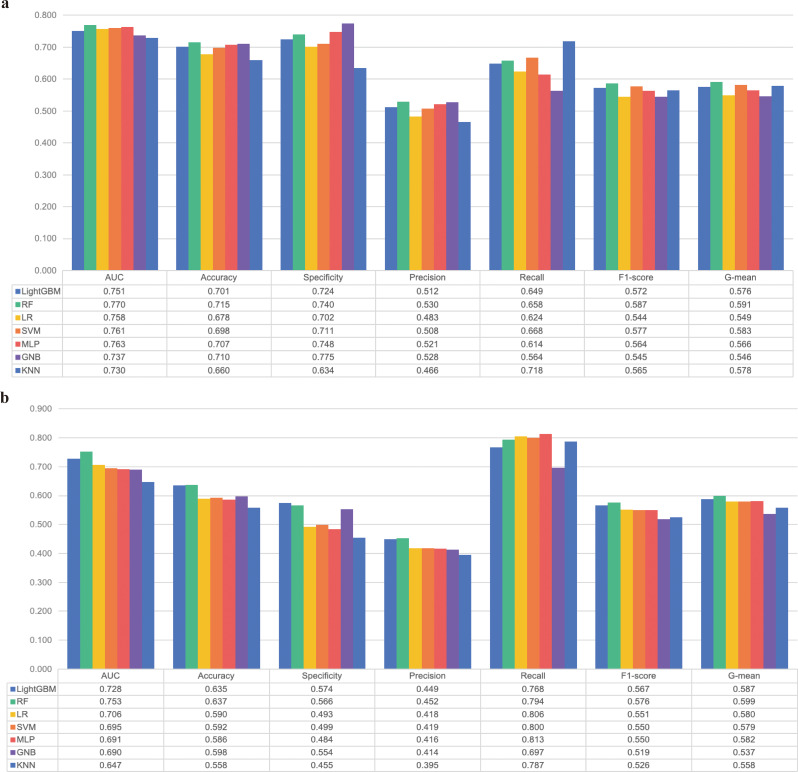
Performance of machine learning models in the validation cohorts. **a**: for internal validation; **b**: for external validation. AUC, area under curve; GNB, Gaussian Naive Bayes; KNN, k-nearest neighbors; LightGBM, light gradient boosting machine; LR, logistic regression; MLP, multilayer perceptron; RF, random forest; SVM, Support Vector Machine

In the *internal validation cohort*, ROC and DCA of the ML models showed that RF had the highest AUC (0.770, 95% CI 0.738–0.802) and the greatest net clinical benefit (Fig. 4a and c). Further calculation of other indicators (Fig. 5a) also revealed that RF had the highest F1 score (0.587) and G mean (0.591).

In the *external validation cohort*, 498 patients with DHF and T2DM were recruited, and their baseline characteristics are shown in Supplementary Table S12. The median age of the external validation cohort was 75.0 years (IQR: 67.0–81.0), and 269 patients (54.0%) were male. RF also had the highest AUC (0.753, 95% CI 0.716–0.791) and the greatest clinical net benefit in the *external validation cohort* (Fig. 4b and d). Further calculations for other indicators (Fig. 5b) also showed that RF had the highest F1 score (0.576) and G mean (0.599).

**Fig. 6:**
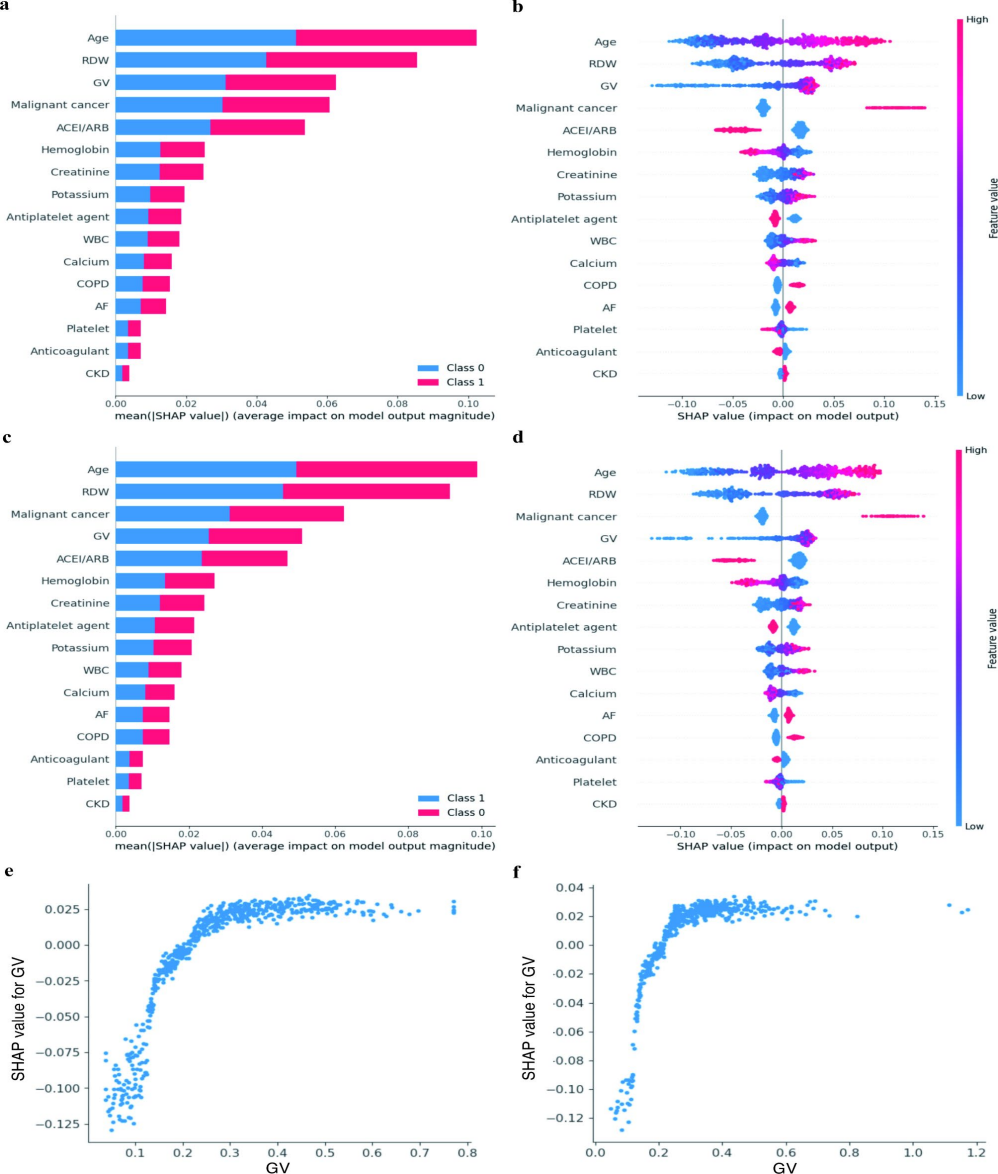
SHAP value and feature importance of random forest in the validation cohorts. **a, b**: SHAP values for all features in internal validation cohort; **c, d**: SHAP values for all features in external validation cohort; **e**: SHAP value for GV in internal validation cohort; **f**: SHAP value for GV in external validation cohort. ACEI, angiotensin converting enzyme inhibitor; ARB, angiotensin receptor blocker; CKD, chronic kidney disease; COPD, chronic obstructive pulmonary disease; GV, glycaemic variability; RDW, red blood cell distribution width; WBC, white blood cell

The order from top to bottom on the Y-axis indicates the feature importance of RF in the internal validation (Fig. 6a and b) and external validation cohorts (Fig. 6c and d). GV was third and fourth in the *internal validation* and *external validation cohorts*, respectively. Figure 6e and f shows that as the GV becomes higher, the greater the positive contribution of GV to the predicted value.

### Mediation analysis

**Fig. 7:**
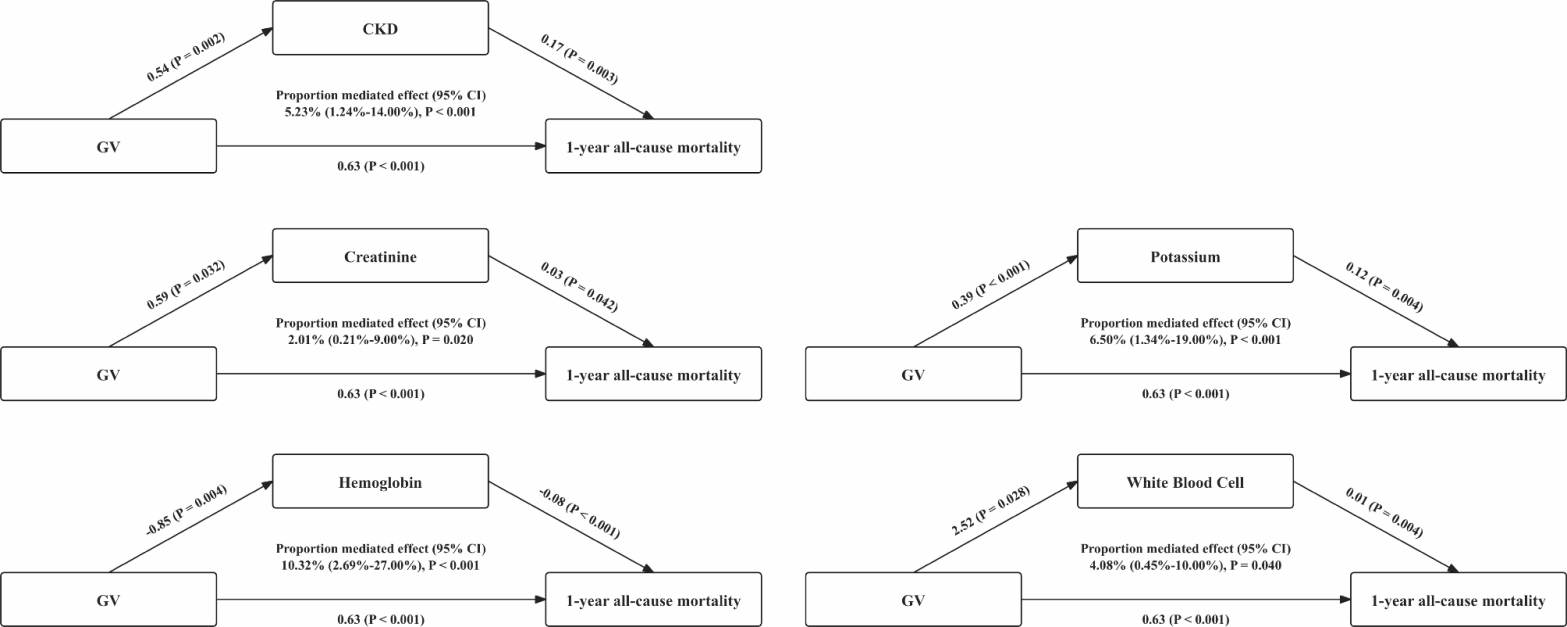
Diagram of mediation effects of GV and 1-year all-cause mortality, CI, confidence interval; CKD, chronic kidney disease; GV, glycaemic variability

The mediation analysis identified significant mediators between the GV and 1-year all-cause mortality, including CKD, creatinine, potassium, hemoglobin, and WBC. Figure 7 shows the results of the mediation effects of the significant mediators. For example, the proportional mediator effect values were 5.23% (95% CI: 1.24-14.00%, *P* < 0.001) for CKD and 10.32% (95% CI: 2.69-27.00%, *P* < 0.001) for hemoglobin. These results emphasise the key role of these mediators in the relationship between TyG and risk of 1-year all-cause mortality in the patients with DHF and T2DM.

## Discussion

Our study utilized the MIMIC-IV database for a retrospective analysis and conducted follow-ups for up to 3 years to evaluate the association between GV and all-cause mortality in individuals with DHF combined with T2DM. The main findings were as following: (i) Higher GV was significantly associated with increased 90-day, 1-year, and 3-year all-cause mortality, suggesting that GV could be used for short-term and long-term mortality risk stratification in patients with DHF and T2DM; (ii) Elevated GV was significantly associated with prolonged hospital stay in patients with DHF and T2DM, indicating that hospital costs and complications may increase; (iii) This analysis revealed that the target of intervention for GV in patients with DHF and T2DM was approximately 25.0%, which could effectively reduce the mortality risk; (iv) GV had the potential to be applied in ML to predict 1-year all-cause mortality in patients with DHF and T2DM, and GV was ranked in the top four contributors in the model.

Our findings align with prior studies, which examined on the effects of GV on patients with CVD. Frisch et al. and Mendez et al. indicated that higher GV was associated with longer hospital stays and increased mortality, independent of factors including age, diabetes, glucose, and HbA1c [27, 28]. Additionally, a meta-analysis involving 76,843 HF patients reported that significant increases in GV during follow-up were linked to a higher risk of mortality (relative risk: 2.18, 95% CI: 1.61–2.96, *P* < 0.001). [28] A United States-based study that monitored blood glucose levels over 72 h, along with dynamic electrocardiogram monitoring in well-controlled T2DM patients with coronary heart disease, confirmed that hypoglycemia and rapid glucose fluctuations were clearly related to myocardial ischemia events [30]. The underlying mechanism may stem from the fact that cells develop some level of adaptability to a consistently high-glucose environment, resulting in relatively stable yet persistent damage to their morphology and function. However, blood glucose fluctuations disrupt this adaptability, with fluctuating hyperglycemia more likely to trigger oxidative stress and promote endothelial cell apoptosis, causing stronger damage to vascular endothelial cells and an increased risk of CVD and their progression [31]. Moreover, in diabetes patients with well-controlled HbA1c levels, short-term GV was positively correlated with the 10-year CVD risk, potentially due to the destabilization of vascular plaques caused by GV [32]. 

Although GV, as assessed by the coefficient of variation from multiple blood glucose measurements, is not yet classified as an independent prognostic risk factor for CVD complications in diabetes, it is increasingly regarded as a key indicator for glucose homeostasis. A GV cut-off of 36.0% has been used to differentiate between stable and unstable diabetes, while maintaining GV below 27.0% has been shown to effectively reduce the risk of hypoglycemia [33]. In our study, we investigated the impact of GV on patients with DHF and T2DM for the first time and observed that a GV threshold of approximately 25.0% related to lower risk of short-term and long-term all-cause mortality, as well as prolonged hospital stays. These findings suggest that tighter GV control may play a key role in improving the prognosis of patients with DHF and T2DM. Furthermore, treatments such as insulin glargine or liraglutide [34], alongside physical activities like low-volume high-intensity interval training and endurance training [35], may help stabilize blood glucose fluctuations effectively. However, our study specifically targeted patients with both HFpEF and T2DM, as this subgroup faces unique metabolic and cardiovascular challenges that may amplify the impact of glycemic variability on outcomes. Expanding the analysis to a broader HFpEF population, including those without T2DM, could provide valuable insights but falls outside the initial scope and objectives of our research. Future studies could explore whether these associations hold in a more general HFpEF cohort and investigate interventions to stabilize glycemic variability in this high-risk group.

Accurately predicting 1-year all-cause mortality in patients with DHF and T2DM is crucial, especially given the high mortality rate in this population. Prausmüller et al. observed that all-cause mortality in patients with DHF and T2DM was approximately twice that of patients with DHF alone (39.2% vs. 26.7%) during a median follow-up of 47 months [36]. In our cohort, the 1-year all-cause mortality rate was 30%, and the 3-year all-cause mortality rate was 38.2%, highlighting the urgency of early risk stratification and intervention for this population. Nevertheless, there is no mature ML prediction model or other validated prediction tool specifically for predicting long-term all-cause mortality in patients with DHF and T2DM.

Although several ML models have been developed for either DHF or T2DM individually, it is unclear whether they can be applied to patients with both diseases. Angraal et al. constructed a RF model for 3-year all-cause mortality in the DHF patients with an AUC of 0.720 (95% CI 0.69–0.75), where admission glucose level was considered one of the significant predictors [37]. Montesanto and colleagues developed a decision-tree model for 5-year all-cause mortality in patients with T2DM with an AUC of 0.73 (95% CI 0.69–0.76), but no glucose-related parameter was included in the model [38]. However, our study specifically developed a machine learning model for predicting 1-year all-cause mortality in patients with DHF and T2DM, which has been externally validated and has superior performance. By addressing the unique needs of this patient population, our model provides a more accurate and reliable tool for early risk stratification and personalised intervention.

Five risk models have been developed for HFpEF: CHARM, MAGGIC, I-Preserve, 3A3B score, and the EMPEROR-Preserved trial. The CHARM model was developed earlier for patients who responded to treatment with candesartan; [39] the MAGGIC model is based on data from multiple clinical trials and observational studies, with the addition of BNP as an important indicator [40], and the I-Preserve study, a randomized controlled trial specifically for patients with HFpEF that evaluated the effect of Irbesartan on patients with HFpEF, with a model of 12 variables, including NT-proBNP, which is more complex and includes quality-of-life scores that are not routinely performed [41]. The 3A3B score is specifically 3 A (Afib, Anemia, Age) and 3B (BMI, Blood pressure, Biomarkers) which are conventional variables [26]. The EMPEROR-Preserved trial enriched the clinical model by adding NT-proBNP and hs-cTnT to the readily available clinical variables, and its mortality risk prediction model had a high c-statistic of 0.79 [42]. Whereas our prediction model was based on the two most common disorders, DHF and T2DM, for this population, combined with the conventional clinical indicators in the previous model, the index of glycemic variability was quoted to enhance the prediction efficacy.

Furthermore, our mediation analyses showed that GV was significantly associated with 1-year all-cause mortality in patients with DHF and T2DM, primarily mediated by these factors CKD, creatinine, potassium, haemoglobin, and WBC. This finding suggests that a significant portion of the mortality risk associated with TyG can be explained by these mediators, highlighting their potential as key targets for intervention.

### Limitations

Our study has several limitations. First, as a single-centre retrospective study, which may limit the generalizability of our findings. Retrospective designs inherently suffer from selection bias, and while we attempted to control for known confounders, unmeasured variables may still impact the results. Second, certain confounding factors that influence blood glucose levels, such as patient diet, daily physical activity, and long-term glycaemic control, were not included in our analysis. This omission may lead to an underestimation of the true impact of GV, as patients could develop additional risk factors during extended follow-up periods. Third, due to the limitations of retrospective data collection, we were unable to include echocardiographic ejection fraction values, which could provide a more precise characterization of heart failure severity. However, given that DHF is characterized by preserved ejection fraction by definition, the absence of this data may not significantly impact the interpretation of our findings. Our study population was clearly defined based on ICD codes for DHF and T2DM, ensuring that the results remain applicable to similar patient populations. Moreover, our inability to obtain the reason for the admission may have resulted in this potential influence not being considered in this analysis. However, we included and adjusted for many other covariates that could have assessed the overall condition of the patients, and these may still have had a different impact on the possible risk of death, although future studies should consider including this variable to more fully assess its impact on the relationship between glycemic volatility and risk of death. Fourth, although our study excluded patients who died within hospital to avoid reverse causality, reliance on existing records also means that changes in clinical practice and patient management over time may have affected the results. Fifth, the absence of real-time monitoring data, such as continuous glucose monitoring, limits our ability to fully capture the time dynamics of blood glucose changes, which could provide a deeper understanding of the role of blood glucose changes in the mortality risk. Sixth, this study did not set a minimum time interval between glucose measurements when calculating glycemic variability, which may introduce bias, particularly in cases where repeated measurements are taken shortly after hypoglycemia. Future studies should consider optimizing data collection strategies to ensure accuracy and consistency in glycemic variability calculations. Finally, The population analysed in this study comprised T2DM with DHF patients who were hospitalized, which limited the generalizability of our findings to the broader T2DM with DHF population. While our results suggest that GV is an important risk factor in these hospitalised patients, further research is needed to confirm if these findings apply to less severe cases, including non-hospitalized patients. The 25% cut-off for GV we identified may also be specific to hospitalised patients, so additional studies are necessary to confirm its relevance across other patient groups. Future research should explore these associations in different care settings and disease severities to validate our findings.

## Conclusion

GV was determined to be an independent risk factor for both short-term and long-term all-cause mortality as well as prolonged length of hospital stay in patients with DHF and T2DM, with a potential intervention threshold identified around 25.0%. This suggests that targeted interventions aimed at controlling GV could play a critical role in reducing mortality risk in this high-risk population. Furthermore, the ML model incorporating GV has a strong predictive ability for 1-year all-cause mortality in patients with DHF and T2DM. Physicians could use this to better identify high-risk patients at an early stage, implement more personalised treatment strategies, thereby improving the long-term survival of patients with DHF and T2DM.

## Electronic supplementary material

Below is the link to the electronic supplementary material.

Supplementary Material 1

## Data Availability

No datasets were generated or analysed during the current study.

## Abbreviations

ACEI: Angiotensin converting enzyme inhibitors
AF: Atrial fibrillation
ARB: Angiotensin receptor blocker
AUC: Area under the curve
BIDMC: Beth Israel Deaconess Medical Centre
CAD: Coronary artery disease
CI: Confidence interval
CKD: Chronic kidney disease
COPD: Chronic obstructive pulmonary disease
CVD: Cardiovascular disease
DCA: Decision curve analysis
DHF: Diastolic heart failure
GV: Glycaemic variability
HF: Heart failure
HR: Hazard ratio
ICD-10: International Classification of Diseases version 10
IQR: Interquartile range
LightGBM: Light gradient boosting machine
MI: Myocardial infarction
MIMIC-IV: Medical Information Mart for Intensive Care IV
ML: Machine learning
OR: Odds ratio
RCS: Restricted cubic spline
RDW: Red blood cell distribution width
RF: Random forest
ROC: Receiver operating characteristic
SHAP: SHapley Additive exPlanations
T2DM: Type 2 diabetes mellitus
WBC: White blood cell count
HbA1c: Glycated Hemoglobin A1c
SD: Standard deviation
VIM: Variance independent of the mean
NRI: Net Reclassification Improvement
IDI: Integrated Discrimination Improvement Index
