# Supplementary material for: Prognostic effects of glycaemic variability on diastolic heart failure and type 2 diabetes mellitus: insights and 1-year mortality machine learning prediction model
Source: Diabetol Metab Syndr. 2024 Nov 23;16:280. doi: 10.1186/s13098-024-01534-2 (PMC11585110; doi:10.1186/s13098-024-01534-2)
Supplement: Supplementary file 1 — Supplementary Material 1 [file 13098_2024_1534_MOESM1_ESM.pdf]

Supplementary Table S1. Proportion of missing values for the variable.

Variables	n (%)
Creatinine	589 (26.99%)
Potassium	587 (26.90%)
Calcium	603 (27.64%)
Hemoglobin	605 (27.73%)
Platelet	605 (27.73%)
Red blood cell distribution width	605 (27.73%)
White blood cell	605 (27.73%)

Supplementary Table S2. Colinearity test of covarites included in Model 4.

Characteristics	Variance inflation factor
Age	1.302
Sex	1.122
Race	1.050
Coronary artery disease	1.196
COPD	1.046
Myocardial infarction	1.131
Atrial fibrillation	1.737
Chronic kidney disease	1.194
Stroke	1.030
Cardiomyopathy	1.039
Dyslipidemia	1.064
Hypertension	1.116
Hepatic disease	1.147
Malignant cancer	1.056
Creatinine	1.499
Potassium	1.209
Calcium	1.124
Hemoglobin	6.960
Platelet	1.291
WBC	1.123
RDW	1.528
Diuretic	1.146
Insulin	1.059
ACEI/ARB	1.123
Beta blocker	1.079
Statin	1.128
Antiplatelet agent	1.264
Anticoagulant	1.629
CABG/PCI	1.089
ICD pacemaker	1.056

Abbreviations: ACEI, angiotensin converting enzyme inhibitor; ARBs, angiotensin receptor blocker; CABG, coronary artery bypass grafting; COPD, chronic obstructive pulmonary disease; ICD, implantable cardioverter defibrillator; PCI, percutaneous coronary intervention; RDW, red blood cell distribution width; WBC, white blood cell count.

Supplementary Table S3. Outcomes of participants by tertiles of glycaemic variability.

Outcomes	ALL	T1	T2	T3	*P*
90-day all-cause mortality, n (%)	366 (16.8)	87 (12.0)	128 (17.6)	151 (20.7)	< 0.001
1-year all-cause mortality, n (%)	672 (30.8)	185 (25.5)	234 (32.2)	253 (34.7)	< 0.001
3-year all-cause mortality, n (%)	834 (38.2)	232 (32.0)	300 (41.3)	302 (41.4)	< 0.001
Hospital length of stay, days	6.79 (4.21, 11.45)	5.71 (3.79, 8.79)	6.88 (4.50, 11.58)	7.98 (4.92, 14.08)	< 0.001
Pronlonged length of hospital stay, n (%)	545 (25.0)	114 (15.7)	184 (25.3)	247 (33.8)	< 0.001

T1: GV ≤ 19.5%, T2: 19.5% < GV ≤ 30.8%, and T3: GV > 30.8%.

Abbreviations: GV, glycaemic variability.

Supplementary Table S4. Association of participants by tertiles of GV and prolonged length of hospital stay.

	Model I*		Model II*		Model III*		Model IV*	
	OR (95% CI)	*P*	OR (95% CI)	*P*	OR (95% CI)	*P*	OR (95% CI)	*P*
T1	*Reference*		*Reference*		*Reference*		*Reference*	
T2	1.68 (1.36-2.07)	< 0.001	1.80 (1.39-2.34)	< 0.001	1.78 (1.36-2.32)	< 0.001	1.67 (1.27-2.21)	< 0.001
T3	2.33 (1.89-2.88)	< 0.001	2.69 (2.09-3.46)	< 0.001	2.66 (2.05-3.44)	< 0.001	2.54 (1.93-3.34)	< 0.001

T1: GV ≤ 19.5%, T2: 19.5% < GV ≤ 30.8%, and T3: GV > 30.8%.

Model 1: unadjusted;

Model 2: adjusted for age, sex and race;

Model 3: based on Model 2 further adjusted for coronary artery disease, chronic obstructive pulmonary disease, myocardial infarction, atrial fibrillation, chronic kidney disease; stroke, cardiomyopathy, dyslipidemia, hypertension, hepatic disease, malignant cancer;

Model 4: based on Model 3 further adjusted for creatinine, potassium, calcium, hemoglobin, platelet, white blood cell, red blood cell distribution width, diuretic, insulin, angiotensin converting enzyme inhibitor/angiotensin receptor blocker, beta blocker, statin, antiplatelet agent, anticoagulant, coronary artery bypass grafting/percutaneous coronary intervention, implantable cardioverter defibrillator pacemaker.

**P for trend* < 0.001 for each model corresponding to this outcome.

Abbreviations: CI, confidence interval; GV, glycaemic variability; OR, odds ratio.

Supplementary Table S5. Comparison of Mortality Discrimination Performance for previous model with or without GV.

	Continuous NRI (95% CI)	P	IDI (95% CI)	P
1-year all-cause mortality				
Previous model	*Reference*		*Reference*	
Previous model + GV	20.4% (2.1%, 38.8%)	0.029	0.8% (0.0%-0.2%)	0.047
3-year all-cause mortality				
Previous model	*Reference*		*Reference*	
Previous model + GV	15.8% (1.9%-33.5%)	0.040	1.0% (1.0%-2.0%)	0.030

Abbreviations: CI, confidence interval; GV, glycaemic variability; NRI, Net Reclassification Improvement; IDI, Integrated Discrimination Improvement Index.

Previous model was defined as: Age ≥ 75 years (2 points); albumin < 3.7 g/dl, anemia, BMI < 22 kg/m^2, NT-proBNP ≥ 1400 pg/ml, BUN ≥ 25 mg/dl (1 point for each).

Supplementary Table S5. Sensitivity analysis for associations between GV with 90-day, 1-year, and 3-year all-cause mortality in post-discharge

		Model I		Model II		Model III		Model IV	
		HR (95% CI)	*P*	HR (95% CI)	*P*	HR (95% CI)	*P*	HR (95% CI)	*P*
90-day all-cause mortality*									
	T1	*Reference*		*Reference*		*Reference*		*Reference*	
	T2	1.31 (0.96, 1.80)	0.090	1.40 (1.02, 1.92)	0.037	1.40 (1.02, 1.93)	0.037	1.29 (0.93, 1.80)	0.124
	T3	1.54 (1.13, 2.09)	0.006	1.77 (1.30, 2.41)	< 0.001	1.81 (1.32, 2.49)	< 0.001	1.79 (1.29, 2.50)	< 0.001
1-year all-cause mortality*									
	T1	*Reference*		*Reference*		*Reference*		*Reference*	
	T2	1.21 (0.98, 1.50)	0.070	1.27 (1.03, 1.57)	0.023	1.27 (1.03, 1.56)	0.028	1.16 (0.93, 1.44)	0.182
	T3	1.26 (1.02, 1.55)	0.029	1.42 (1.15, 1.75)	0.001	1.42 (1.15, 1.75)	0.001	1.35 (1.08, 1.69)	0.008
3- year all-cause mortality*									
	T1	*Reference*		*Reference*		*Reference*		*Reference*	
	T2	1.29 (1.07, 1.55)	0.006	1.35 (1.13, 1.63)	0.001	1.33 (1.10, 1.60)	0.003	1.25 (1.03, 1.51)	0.022
	T3	1.26 (1.05, 1.52)	0.014	1.41 (1.17, 1.70)	< 0.001	1.38 (1.14, 1.67)	< 0.001	1.34 (1.10, 1.64)	0.004

T1: GV ≤ 18.9%, T2: 18.9% < GV ≤ 30.6%, and T3: GV > 30.6%.

Model 1: unadjusted;

Model 2: adjusted for age, sex and race;

Model 3: based on Model 2 further adjusted for coronary artery disease, chronic obstructive pulmonary disease, myocardial infarction, atrial fibrillation, chronic kidney disease; stroke, cardiomyopathy, dyslipidemia, hypertension, hepatic disease, malignant cancer;

Model 4: based on Model 3 further adjusted for creatinine, potassium, calcium, hemoglobin, platelet, white blood cell, red blood cell distribution width, diuretic, insulin, angiotensin converting enzyme inhibitor/angiotensin receptor blocker, beta blocker, statin, antiplatelet agent, anticoagulant, coronary artery bypass grafting/percutaneous coronary intervention, implantable cardioverter defibrillator pacemaker.

**P for trend* < 0.001 for each model corresponding to this outcome.

Abbreviations: CI, confidence interval; GV, glycaemic variability; HR, hazard ratio.

Supplementary Table S7. Sensitivity analysis with the addition of other blood glucose indicators.

		Model	
		HR (95% CI)	*P*
90-day all-cause mortality			
	T1	*Reference*	
	T2	2.52 (0.91-6.99)	0.077
	T3	3.00 (1.13-7.95)	0.028
1-year all-cause mortality			
	T1	*Reference*	
	T2	1.63 (0.87-3.06)	0.126
	T3	1.92 (1.01-3.63)	0.046
3- year all-cause mortality			
	T1	*Reference*	
	T2	1.75 (1.02-3.02)	0.043
	T3	1.79 (1.02-3.15)	0.044

T1: GV ≤ 19.5%, T2: 19.5% < GV ≤ 30.8%, and T3: GV > 30.8%.

Model: adjusted for age, sex, race, coronary artery disease, chronic obstructive pulmonary disease, myocardial infarction, atrial fibrillation, chronic kidney disease, stroke, cardiomyopathy, dyslipidemia, hypertension, hepatic disease, malignant cancer, creatinine, potassium, calcium, hemoglobin, platelet, white blood cell, red blood cell distribution width, fasting blood glucose, mean glucose, glycated hemoglobin A1c, diuretic, insulin, angiotensin converting enzyme inhibitor/angiotensin receptor blocker, beta blocker, statin, antiplatelet agent, anticoagulant, coronary artery bypass grafting/percutaneous coronary intervention, implantable cardioverter defibrillator pacemaker.

Abbreviations: CI, confidence interval; GV, glycaemic variability; HR, hazard ratio.

Supplementary Table S8. Sensitivity analysis after excluding malignant cancer patients.

		Model	
		HR (95% CI)	*P*
90-day all-cause mortality*			
	T1	*Reference*	
	T2	1.63 (1.17-2.27)	0.004
	T3	2.02 (1.45-2.83)	< 0.001
1-year all-cause mortality*			
	T1	*Reference*	
	T2	1.44 (1.15-1.81)	0.002
	T3	1.69 (1.34-2.14)	< 0.001
3- year all-cause mortality*			
	T1	*Reference*	
	T2	1.50 (1.22-1.83)	< 0.001
	T3	1.63 (1.33-2.01)	< 0.001

T1: GV ≤ 19.5%, T2: 19.5% < GV ≤ 30.8%, and T3: GV > 30.8%.

Model: adjusted for age, sex, race, coronary artery disease, chronic obstructive pulmonary disease, myocardial infarction, atrial fibrillation, chronic kidney disease, stroke, cardiomyopathy, dyslipidemia, hypertension, hepatic disease, creatinine, potassium, calcium, hemoglobin, platelet, white blood cell, red blood cell distribution width, diuretic, insulin, angiotensin converting enzyme inhibitor/angiotensin receptor blocker, beta blocker, statin, antiplatelet agent, anticoagulant, coronary artery bypass grafting/percutaneous coronary intervention, implantable cardioverter defibrillator pacemaker.

**P for trend* < 0.001 for each model corresponding to this outcome.

Abbreviations: CI, confidence interval; GV, glycaemic variability; HR, hazard ratio.

Supplementary Table S9. Associations between SD and VIM with 90-day, 1-year, and 3-year all-cause mortality.

		SD		VIM	
		HR (95% CI)	*P*	HR (95% CI)	*P*
90-day all-cause mortality*					
	T1	*Reference*		*Reference*	
	T2	1.70 (1.28-2.26)	< 0.001	1.49 (1.13-1.96)	0.005
	T3	2/04 (1.52-2.73)	< 0.001	1.61 (1.22-2.13)	< 0.001
1-year all-cause mortality*					
	T1	*Reference*		*Reference*	
	T2	1.43 (1.17-1.75)	< 0.001	1.28 (1.05-1.56)	0.015
	T3	1.60 (1.30-1.97)	< 0.001	1.45 (1.19-1.77)	< 0.001
3- year all-cause mortality*					
	T1	*Reference*		*Reference*	
	T2	1.40 (1.17-1.68)	< 0.001	1.31 (1.10-1.56)	0.003
	T3	1.52 (1.26-1.83)	< 0.001	1.38 (1.16-1.65)	< 0.001

SD: T1: ≤ 26.2mg/dl, T2: 26.2mg/dl < SD ≤ 50.7mg/dl, T3: > 50.7mg/dl.
VIM: T1: ≤ 0.016%, T2: 0.016% < VIM ≤ 0.025%, T3: > 0.025%.
SD of blood glucose was calculated to assess glycemic variability during the hospital stay, reflecting the average deviation of glucose measurements from the mean value.
VIM was calculated using the formula: VIM = SD / Mean^ 1.5, where the exponent 1.5 was chosen to standardize variability across different mean blood glucose.
Adjusted for age, sex, race, coronary artery disease, chronic obstructive pulmonary disease, myocardial infarction, atrial fibrillation, chronic kidney disease, stroke, cardiomyopathy, dyslipidemia, hypertension, hepatic disease, malignant cancer, creatinine, potassium, calcium, hemoglobin, platelet, white blood cell, red blood cell distribution width, diuretic, insulin, angiotensin converting enzyme inhibitor/angiotensin receptor blocker, beta blocker, statin, antiplatelet agent, anticoagulant, coronary artery bypass grafting/percutaneous coronary intervention, implantable cardioverter defibrillator pacemaker.
**P for trend* < 0.001 for each model corresponding to this outcome.
Abbreviations: CI, confidence interval; HR, hazard ratio; SD, standard deviation; VIM, variance independent of the mean.

Supplementary Table S10. Associations between GV divided by 25.0 % with 90-day, 1-year, and 3-year all-cause mortality

		HR/OR (95% CI)	*P*
Whole primary analysis cohort			
90-day all-cause mortality	G1	*Reference*	
	G2	1.54 (1.24, 1.91)	< 0.001
1-year all-cause mortality	G1	*Reference*	
	G2	1.37 (1.17, 1.61)	< 0.001
3- year all-cause mortality	G1	*Reference*	
	G2	1.34 (1.16, 1.55)	< 0.001
Prolonged length of hospital stay	G1	*Reference*	
	G2	1.80 (1.45, 2.23)	< 0.001
Post-discharge of primary analysis cohort			
90-day all-cause mortality	G1	*Reference*	
	G2	1.47 (1.13, 1.90)	0.004
1-year all-cause mortality	G1	*Reference*	
	G2	1.27 (1.06, 1.51)	0.008
3- year all-cause mortality	G1	*Reference*	
	G2	1.27 (1.08, 1.48)	0.003

G1: GV < 25.0%, G2: GV ≥ 25.0%.

Cox proportional hazards models adjusted for age, sex, race, coronary artery disease, chronic obstructive pulmonary disease, myocardial infarction, atrial fibrillation, chronic kidney disease; stroke, cardiomyopathy, dyslipidemia, hypertension, hepatic disease, malignant cancer, creatinine, potassium, calcium, hemoglobin, platelet, white blood cell, red blood cell distribution width, diuretic, insulin, angiotensin converting enzyme inhibitor/angiotensin receptor blocker, beta blocker, statin, antiplatelet agent, anticoagulant, coronary artery bypass grafting/percutaneous coronary intervention, implantable cardioverter defibrillator pacemaker.

Abbreviations: CI, confidence interval; GV, glycaemic variability; HR, hazard ratio; OR, odds ratio.

Supplementary Table S11. Best hyperparameters of each classifier.

Classifiers	Hyperparameters	
Light gradient boosting machine	n_estimators	154
	max_depth	5
	learning_rate	0.01
	boosting_type	gbdt
	objective	binary
	num_leaves	31
	min_child_samples	146
	class_weight	'balanced'
Random Forest	n_estimators	165
	max_depth	5
	criterion	'gini'
	min_samples_leaf	31
	random_state	43
	class_weight	'balanced'
	min_samples_split	2
Logistic Regression	C	0.1
	penalty	l2
	solver	'liblinear'
	max_iter	100
	class_weight	'balanced'
Support Vector Machine	C	2
	kernel	'rbf'
	probability	True
	tol	0.0001
	class_weight	'balanced'
Multilayer Perceptron	solver	'adam'
	activation	'relu'
	hidden_layer_sizes	[5,10]
	alpha	0.011
	max_iter	200
	random_state	4
Gaussian Naive Bayes	var_smoothing	1e-09
K-nearest Neighbors	n_neighbors	28
	p	2
	metric	manhattan
	weights	distance

Supplementary Table S12. Descriptions of patients in the *external validation cohort*.

	ALL (N = 498)	1-year survivors (N = 343)	1-year non-survivors (N = 155)	*P*
Age, years	75.00 (67.00, 81.00)	78.00 (58.00, 87.00)	77.00 (68.00, 84.00)	0.001
Male, n (%)	269 (54.0)	189 (55.1%)	80 (51.6%)	0.497
Comorbidities, n (%)				
COPD	120 (24.1%)	72 (21.0%)	48 (31.0%)	0.018
Atrial fibrillation	246 (49.4%)	168 49.0%)	78 (50.3%)	0.847
Chronic kidney disease	291 (58.4%)	185 (63.6%)	106 (68.4%)	0.003
Malignant cancer	67 (13.5%)	31 (9.0%)	36 (23.2%)	< 0.001
Laboratory results				
Creatinine, mg/dL	1.40 (1.00, 2.30)	1.70 (1.07, 3.12)	1.50 (1.10, 2.50)	0.010
Potassium, mmol/L	4.20 (3.90, 4.70)	4.10 (3.78, 4.60)	4.30 (3.90, 4.80)	0.113
Calcium, mg/dL	8.90 (8.50, 9.30)	8.70 (8.40, 8.92)	8.90 (8.40, 9.40)	0.689
Hemoglobin, g/L	9.95 (8.50, 11.70)	8.85 (7.40, 10.95)	9.40 (8.30, 10.90)	< 0.001
Platelet, ×10^9/L	212.50 (162.00, 282.25)	194.00 (153.75, 263.00)	213.00 (150.00, 283.00)	0.409
White blood cell, ×10^9/L	8.60 (6.40, 12.20)	7.45 (5.38, 11.53)	9.80 (7.10, 13.50)	0.003
RDW, %	15.00 (14.00, 17.00)	15.45 (14.18, 17.43)	16.00 (14.60, 17.60)	< 0.001
Treatments, n (%)				
ACEI/ARB	104 (20.9%)	84 (24.5%)	20 (12.9%)	0.003
Antiplatelet agent	186 (37.3%)	137 (39.9%)	49 (31.6%)	0.089
Anticoagulant	92 (18.5%)	73 (21.3%)	19 (12.3%)	0.018
Length of hospital stay, days	8.31 (4.87, 15.64)	8.56 (4.50, 16.72)	10.00 (5.50, 17.63)	0.007
Glycaemic variability	0.28 (0.20, 0.37)	0.28 (0.20, 0.38)	0.28 (0.21, 0.46)	0.655

Abbreviations: ACEI, angiotensin converting enzyme inhibitor; ARB, angiotensin receptor blocker; COPD, chronic obstructive pulmonary disease; RDW, red blood cell distribution width.

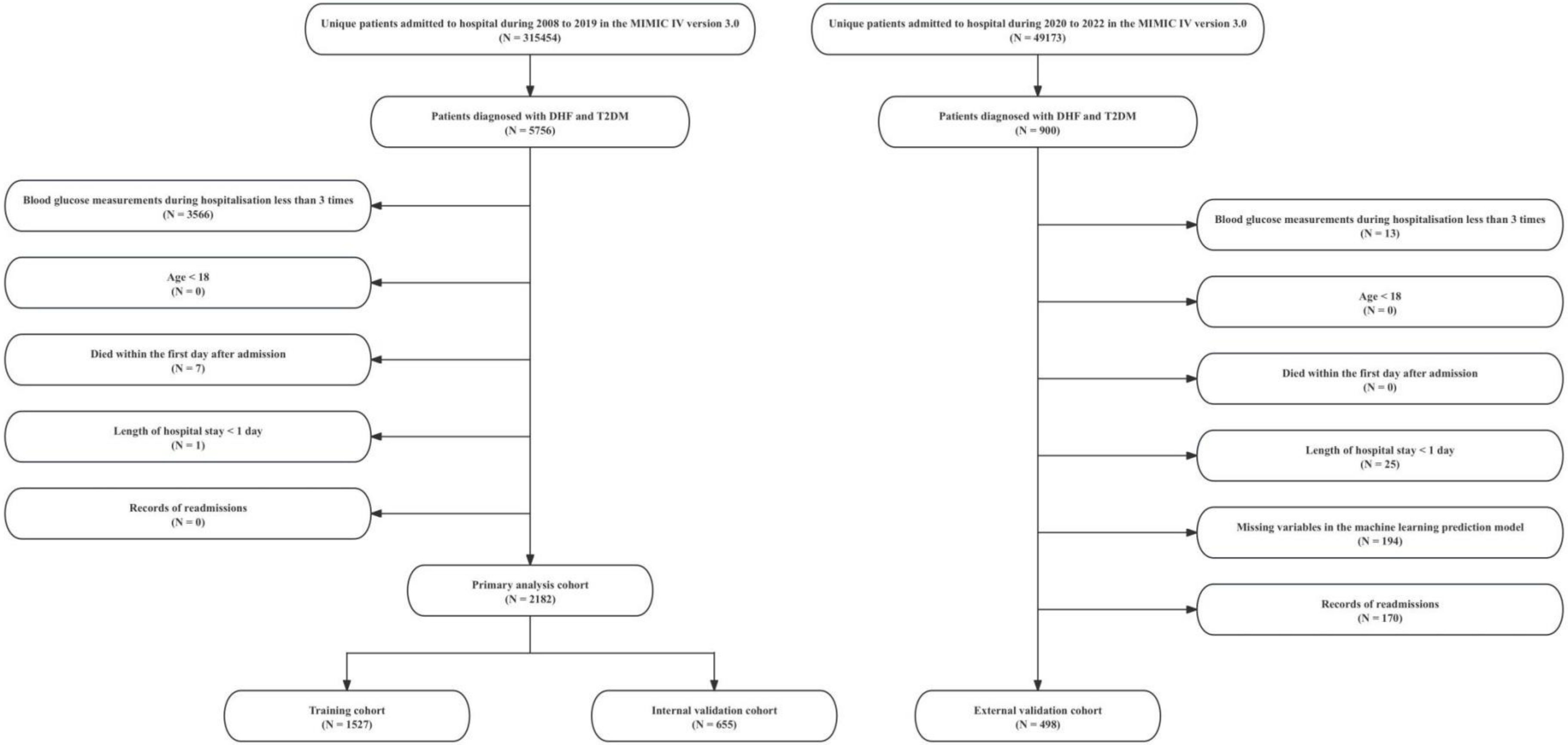

Supplementary Fig. S1 Flowchart of this study. HFpEF, heart failure with preserved ejection fraction; MIMIC, Medical Information Mart for Intensive Care; T2DM, type 2 diabetes mellitus.

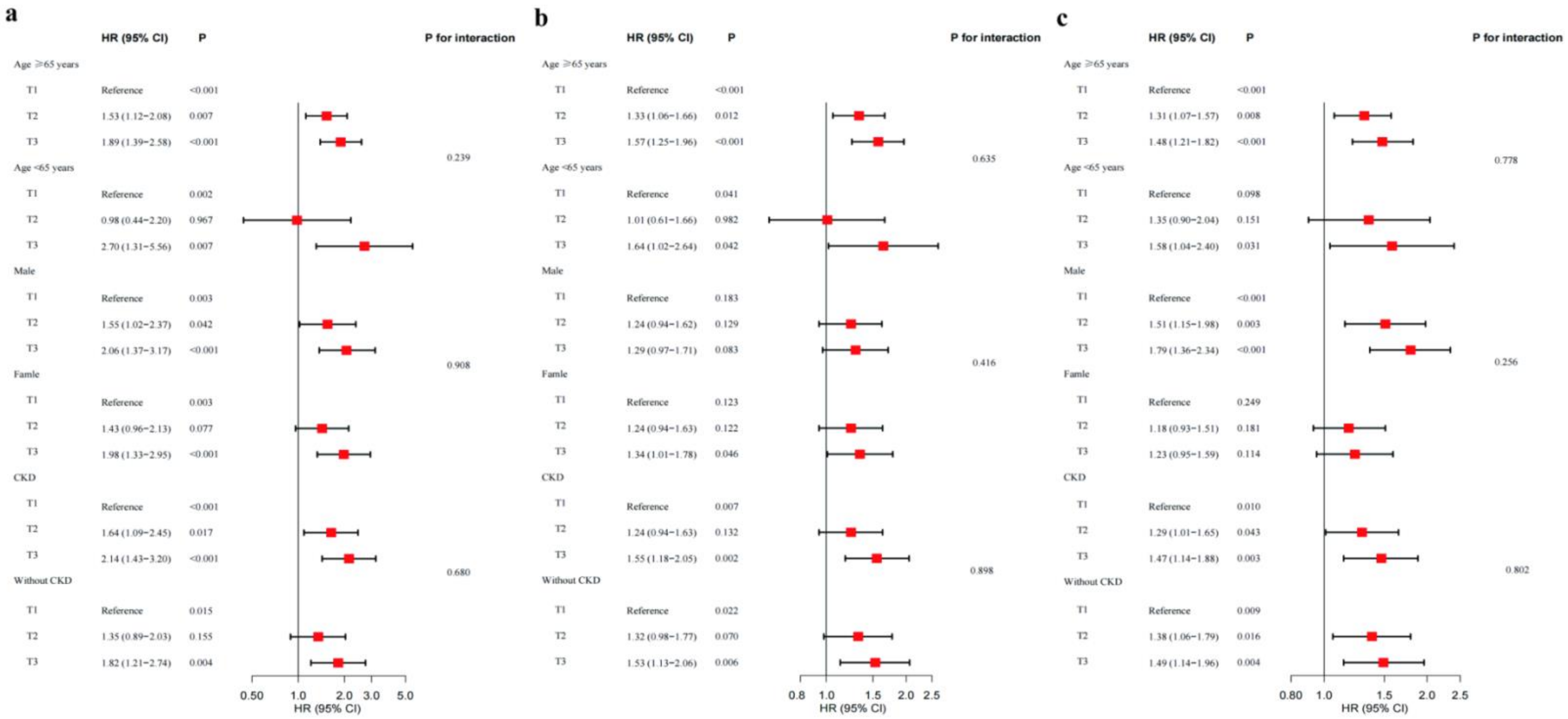

Supplementary Fig. S2 Subgroup analyses for associations between GV with 90-day (a), 1-year (b), and 3-year (c) all-cause mortality. T1: GV ≤ 19.5%, T2: 19.5% < GV ≤ 30.8%, and T3: GV > 30.8%. Adjusted for age, sex, race, coronary artery disease, chronic obstructive pulmonary disease, myocardial infarction, atrial fibrillation, chronic kidney disease, stroke, cardiomyopathy, dyslipidemia, hypertension, hepatic disease, malignant cancer, creatinine, potassium, calcium, hemoglobin, platelet, white blood cell, red blood cell distribution width, diuretic, insulin, angiotensin converting enzyme inhibitor/angiotensin receptor blocker, beta blocker, statin, antiplatelet agent, anticoagulant, coronary artery bypass grafting/percutaneous coronary intervention, implantable cardioverter defibrillator pacemaker. CI, confidence interval; CKD, chronic kidney disease; GV, glycaemic variability; HR, hazard ratio.

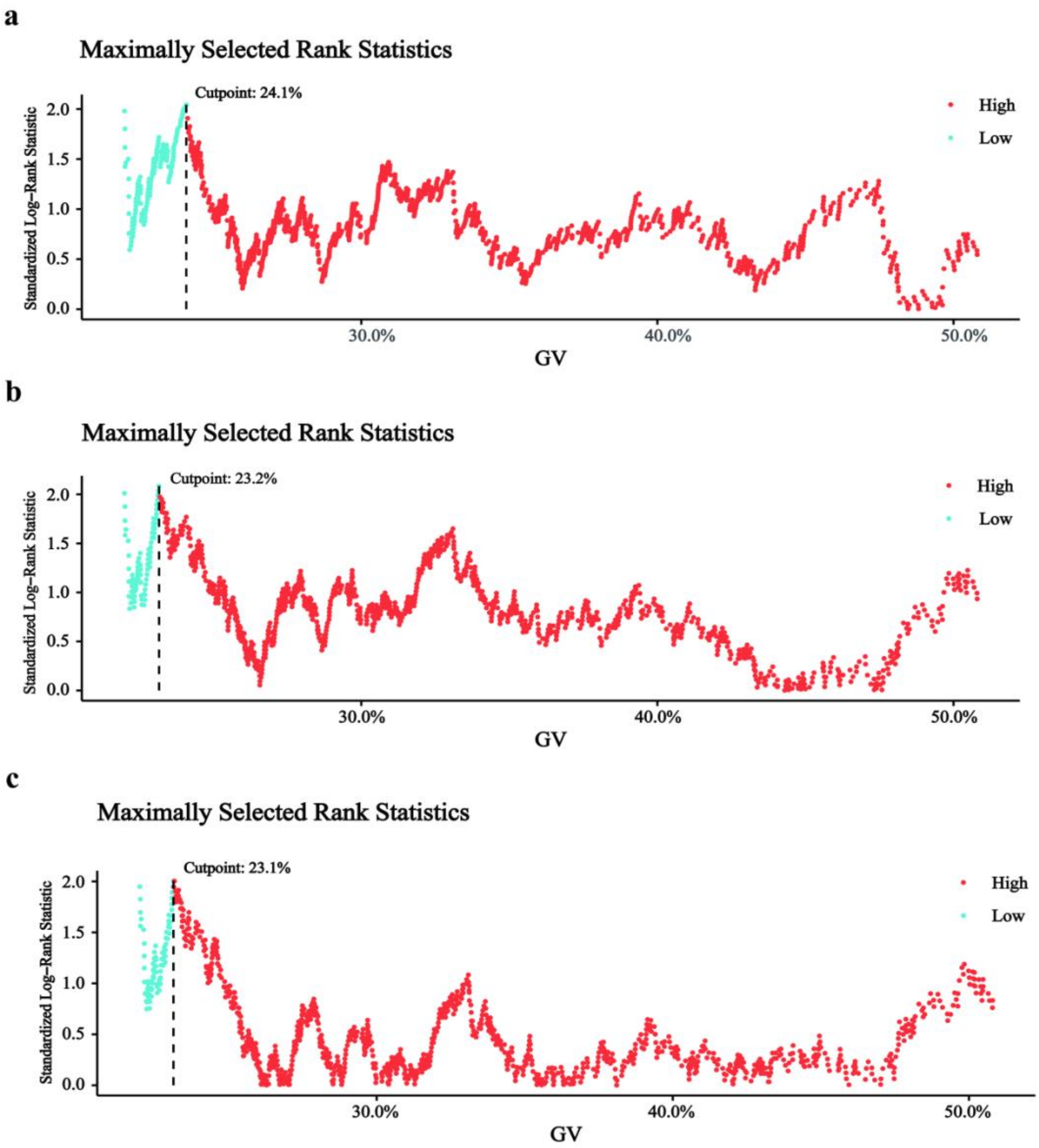

Supplementary Fig. S3 Visualisation of the optimal GV cut-off points for 90-day (a), 1-year (b), and 3-year (c) all-cause mortality. GV, glycaemic variability.

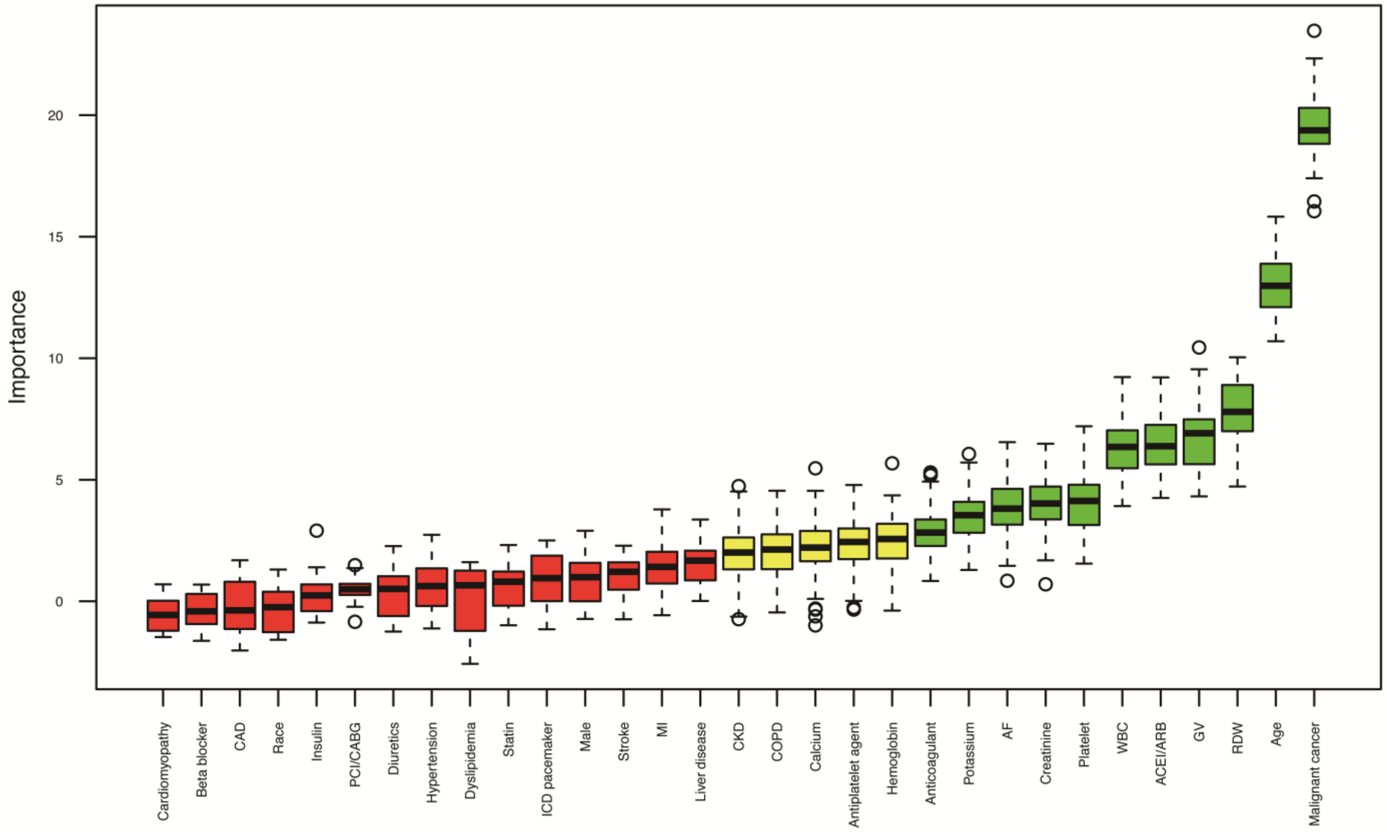

Supplementary Fig. S4 Boruta feature selection for predicting 1-year all-cause mortality. ACEI, angiotensin converting enzyme inhibitor; AF, atrial fibrillation; ARBs, angiotensin receptor blocker; CABG, coronary artery bypass grafting; CAD, coronary artery disease; CKD, chronic kidney disease; COPD, chronic obstructive pulmonary disease; GV, glycaemic variability; ICD, implantable cardioverter defibrillator; PCI, percutaneous coronary intervention; RDW, red blood cell distribution width; WBC, white blood cell.

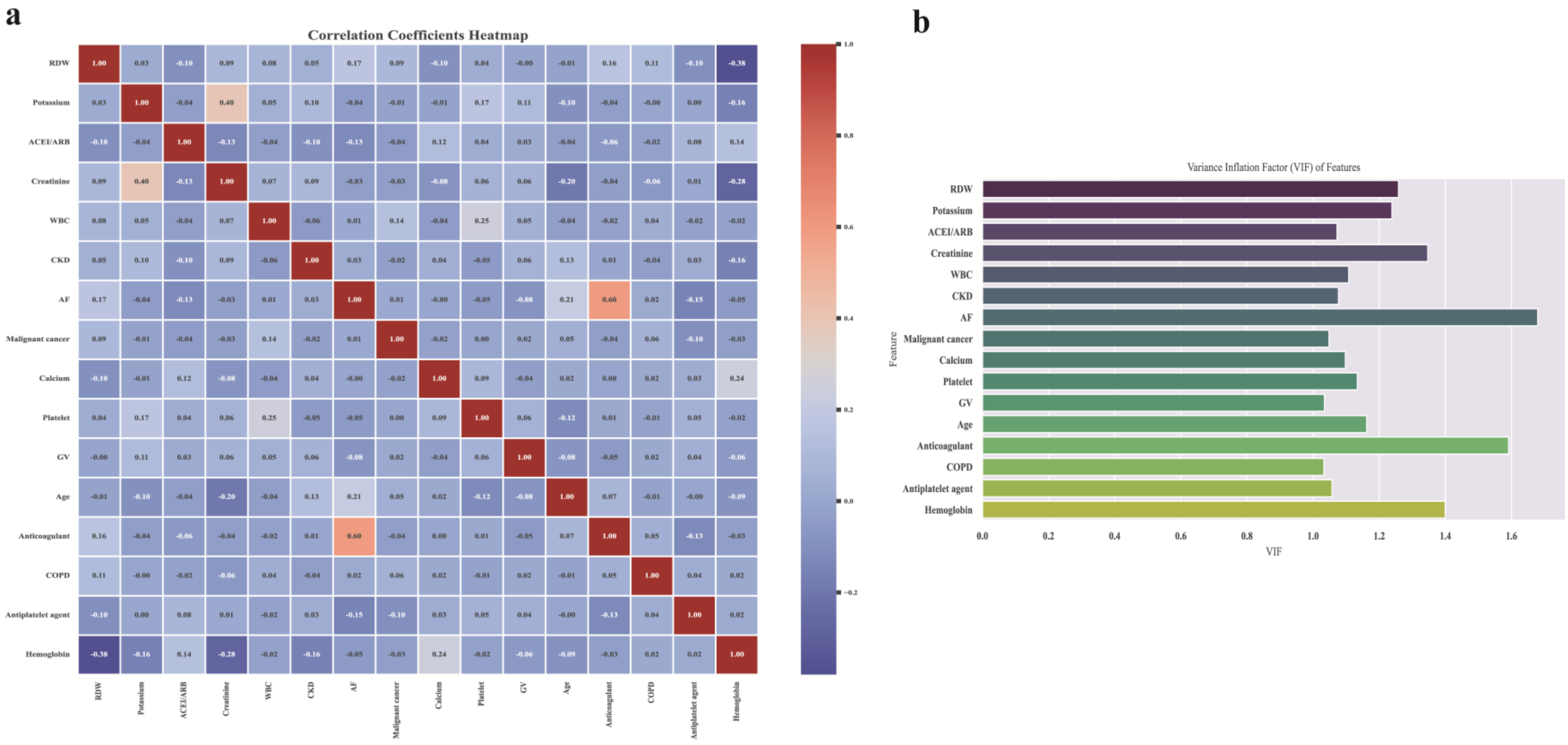

Supplementary Fig. S5. Pearson's correlation test and variance inflation factor test among selected features. ACEI, angiotensin converting enzyme inhibitor; AF, atrial fibrillation; ARBs, angiotensin receptor blocker; CABG, coronary artery bypass grafting; CAD, coronary artery disease; CKD, chronic kidney disease; COPD, chronic obstructive pulmonary disease; GV, glycaemic variability; ICD, implantable cardioverter defibrillator; PCI, percutaneous coronary intervention; RDW, red blood cell distribution width; WBC, white blood cell.
